# A RANKL mutant used as an inter-species vaccine for efficient immunotherapy of osteoporosis

**DOI:** 10.1038/srep14150

**Published:** 2015-09-28

**Authors:** Changzhen Liu, Yunfeng Zhao, Wen He, Wei Wang, Yuan Chen, Shiqian Zhang, Yijing Ma, Jin Gohda, Takaomi Ishida, Thomas S. Walter, Raymond J. Owens, David I. Stuart, Jingshan Ren, Bin Gao

**Affiliations:** 1 Beijing Key Laboratory of Research of Chinese Medicine on Prevention and Treatment for Major Diseases, Experimental Research Center, China Academy of Chinese Medical Sciences, NO.16, Dongzhimennei South Street, Dongcheng District, Beijing 100700, China; 2 CAS Key Laboratory of Pathogenic Microbiology and Immunology, Institute of Microbiology, Chinese Academy of Sciences, 1 Beichen Xilu, Beijing 100101, China; 3 Research Center for Asian Infectious Diseases, Institute of Medical Science, The University of Tokyo, 4-6-1 Shirokanedai Minato-ku, Tokyo 108-8639, Japan; 4 Division of Structural Biology, The Wellcome Trust Centre for Human Genetics, The Henry Welcome Building for Genomic Medicine, University of Oxford, Roosevelt Drive, Oxford, OX3 7BN, UK; 5 Oxford Protein Production Facility UK, Research Complex at Harwell, Rutherford Appleton Laboratory Harwell, Science and Innovation Campus, Oxfordshire, OX11 0FA, UK; 6 Abingen Ltd, Oxford, UK

## Abstract

Anti-cytokine therapeutic antibodies have been demonstrated to be effective in the treatment of several auto-immune disorders. However, The problems in antibody manufacture and the immunogenicity caused by multiple doses of antibodies inspire people to use auto-cytokine as immunogen to induce anti-cytokine antibodies. Nevertheless, the tolerance for inducing immune response against self-antigen has hindered the wide application of the strategy. To overcome the tolerance, here we proposed a strategy using the inter-species cytokine as immunogen for active immunization (TISCAI) to induce anti-cytokine antibody. As a proof of concept, an inter-species cytokine RANKL was successfully used as immunogen to induce anti-RANKL immune response. Furthermore, to prevent undesirable side-effects, the human RANKL was mutated based on the crystal structure of the complex of human RANKL and its rodent counterpart receptor RANK. We found, the antibodies produced blocked the osteoclast development *in vitro* and osteoporosis in OVX rat models. The results demonstrated this strategy adopted is very useful for general anti-cytokine immunotherapy for different diseases settings.

In recent years, anti-cytokine immunotherapy has revolutionized the treatment of many chronic diseases associated with abnormal cytokine production^1^,^2^,^3^. Studies on passive anti-cytokine immunotherapy with specific high-affinity antibodies were performed in animal models and/or clinical trials for various disease settings, *e.g.*, rheumatoid arthritis, multiple sclerosis, inflammatory bowel disease, asthma, Crohn’s disease, psoriasis and other articular autoimmune disorders^2^,^4^. TNF-alpha antibodies have emerged as a novel class of drugs for several auto-immune disorders^5^. IL-6 receptor-specific anti-cytokine antibodies have been demonstrated to be effective against rheumatoid arthritis^6^ and juvenile idiopathic arthritis^7^. Recently, Denosumab (Prolia), a fully humanized anti-RANKL monoclonal antibody, has been approved by the FDA to treat osteoporosis^8^ and bone loss in patients with prostate or breast cancer undergoing hormone ablation therapy^9^.

Despite its medical and commercial success, passive anti-cytokine immunotherapy has several drawbacks: the difficulty and high costs of production, the compliance required from patients for regular infusions, and the hampered efficacy of anti-cytokine biologics by their limited half-life and immunogenicity caused by multiple dosages. An alternative solution that could potentially overcome these drawbacks is active anti-cytokine immunotherapy^10^,^11^. By this strategy, anti-auto-cytokine antibodies are produced by the immune system of the body as a result of active vaccination. However, to produce the desired antibody response against a self-protein immune tolerance needs to be broken. The common anti-cytokine vaccines are prepared from self-protein converted into derivatives devoid of biological activity after treatment with glutaraldehyde or formaldehyde^12^. The inactive derivatives could be coupled to carrier proteins, such as keyhole limpet hemocyanin (KLH) to prepare immunocomplex conjugate^13^. At present, several conjugates including TNF-α, against psoriasis (Phase I–IIA) and Crohn’s disease (Phase I), and IFN-α, against AIDS (EURIS trial Phase II–III), are undergoing clinical trials. Other carriers with strong immunogenicity such as virus-like particles of the bacteriophage Qβ (VLP-Qβ)^14^,^15^, tetanus toxoid or P64K protein from *Neisseria meningitidis*^16^, and ovalbumin^17^ are also being investigated. Another way to break the self-tolerance is to incorporate with the cytokine helper T-epitopes such as those from ovalbumin sequence or egg-white lysozyme sequence^18^. DNA vaccinations with genes coding the pathogenic cytokines were successfully used to prevent collagen-induced arthritis, experimental autoimmune encephalomyelitis, and spontaneous lupus-like autoimmune disease in a variety of animal models^19^,^20^,^21^. However, modification of auto-protein changes the structure so that the antibodies generated may fail to recognize the original cytokine, furthermore, immune response to the carrier might generate undesired side-effects.

In this study, we tested a new strategy in which an inter-species cytokine is used as an immunogen to induce anti-cytokine response to block undesired cytokine effects. To be the inter-species cytokine immunogen must meet two criteria: 1, the immunogen must have a very similar structure to the inter-species cytokine to be neutralized; 2, immunogen itself should not have any biological activities. Therefore, we will identify a cytokine from related species which has ability to bind to correspondent receptor but use it as an immunogen only after it has been mutated to ablate receptor binding activity.

RANKL (the receptor activator of the nuclear factor kappa-B ligand, also named as TNFRSF11, TRANCE and ODF) and its receptor RANK (the receptor activator of the nuclear factor kappa-B) are essential for the development and activation of osteoclasts, and therefore play important roles in regulating bone remodeling^22^,^23^. RANKL is a member of the tumor necrosis factor (TNF) cytokine family and was cloned by four different groups^24^,^25^,^26^,^27^. It is a type II transmembrane protein found primarily on the surface of osteoblasts, activated T-cells and bone marrow stromal cells^28^. Human and mouse RANKL (hRANKL and mRANKL) share 87% identity in protein sequence indicating that this protein is highly evolutionary conserved. The functional receptor for RANKL, RANK, is a member of the TNF receptor (TNFR) family. It is a type I transmembrane protein, consisting of around 620 amino acids with ∼85% identity between mouse and human homologs^24^. The N-terminal extracellular region from position 30 to 194 is composed of four tandem cysteine-rich pseudo repeat sequences (CRDs) that are characteristic of the TNF receptor family. These repeated CRDs confer an elongated shape and the ligand contact area upon the receptor. When interaction with the ligands, three separated RANK extracellular regions bind along the clefts formed between neighboring monomers of a RANKL homotrimer, leading to the assembly of three RANK cytoplasmic regions^29^. The cytoplasmic region of RANK consisting of about 383 amino acid residues is one of the longest cytoplasmic domains in the TNF receptor family. Like other members of the family this region lacks intrinsic enzymatic activity, and transduces intracellular signals by recruiting various adaptor proteins including TNF receptor–associated factors (TRAFs), leading to the activation of NF-κB, JNK, ERK, p38, NFATc1, and Akt signaling pathways^30^,^31^,^32^. Osteoprotegerin (OPG), the soluble homolog of RANK, is a natural decoy receptor of RANKL^33^. It is primarily secreted by bone marrow stromal cells and osteoblasts and acts as a key endogenous regulator of the RANKL–RANK pathway by blocking the binding of RANKL to RANK. The important effect of OPG has been demonstrated in *OPG* knockout mice which exhibited severe osteoporosis^34^,^35^. It has been demonstrated that the ratio of RANKL/OPG represents an important determinant of bone resorption in several disease models and settings^36^. Thus, RANKL, RANK and OPG provide a ligand/receptor/receptor antagonist system for controlling bone homeostasis and other related biological processes.

Since Denosumab is as effective as bisphosphonates which are the current standard of care for osteoporosis and other bone resorption diseases^8^, an active anti-RANKL immunotherapy would be a viable strategy for the treatment of osteoporosis and other bone related diseases. In consideration of ~90% identity of the RANKL ectodomain (residues 162–317 of hRANKL, and residues 161–316 of mRANKL), and successful crystallization of a complex of mouse RANK (mRANK) and hRANKL, we speculated that using hRANKL ectodomain as immunogen would induce the anti-RANKL antibodies to neutralize endogenous RANKL in rodent animals. The high level of similarity should provide both immunogenicity of injected exogenous protein and neutralizing ability of the generated anti-RANKL antibodies for endogenous RANKL. To demonstrate an inter-species cytokine as vaccine we tested hRANKL as an immunogen for the treatment of osteoporosis in a rodent model. Meanwhile, to prevent the possible side-effects of a cytokine vaccine, we set out to create hRANKL mutants lost the binding activity to a rodent RANK. The results showed, with the help of the structure of hRANKL-mRANK complex we identified several hRANKL mutants that bound poorly to mRANK but stimulate an antibody response that neutralize RANKL in rats and inhibit osteoclast formation *in vitro*. The application of hRANKL mutants as vaccine successfully prevents the osteoporosis in ovariectomized (OVX) rats.

## Result

### The structure of hRANKL-mRANK complex

We have recently reported the crystal structure of mRANK ectodomain in complex with mRANKL ectodomain^29^. To determine the complex structure of hRANKL and mouse receptor both recombinant hRANKL and mRANK were expressed and purified with similar methodology. We failed to obtain any complex crystal although many different crystallization conditions were screened. A crystal of mRANK/mRANKL was crushed and seeded into a solution containing purified mRANK and hRANKL and crystals were obtained. The crystals diffracted to 2.8 Å using synchrotron radiation and the structure of the complex was determined using molecular replacement with an initial model of mRANKL-mRANK^29^. The final refined model contains residues of 35–199 of mRANK and 162–317 of hRANKL.

**Figure 1:**
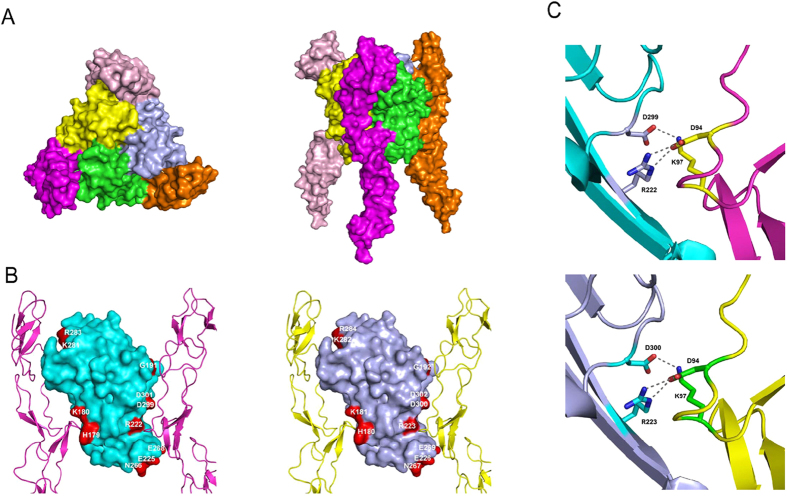
Structure of hRANKL–mRANK complex. (**A**) Overall structures of the complex. Three hRANKL molecules colored yellow, green and light blue form a homotrimer, and three mRANK monomers colored purple, orange and light pink bind to the grooves of hRANKL trimer individually. (**B**) Comparasion of key residues in RANKL involved in the two forms of ligand-receptor interactions. *Left*, Open book view of mRANKL–mRANK complex (PDB code 3ME2); *Right*, Open book view of hRANKL–mRANK complex. The key residues in RANKL involved in interactions were colored red. (**C**) Close-up view of a contact region between ligand and receptor. *Up*, the region lies in mRANKL–mRANK complex; *Down*, the region lies in hRANKL–mRANK complex.

Similar to the complexes of mRANKL-mRANK and other classical TNF-TNFR family members, hRANKL and mRANK form a biological hetero-hexameric complex with one receptor binding along each of the three clefts between neighboring monomers of the ligand homotrimer (Fig. 1A). The 90% identity of RANKL ectodomain (residues 162–317 of hRANKL and residues 161–316 of mRANKL) between *Homo sapiens* and *Mus musculus* indicates, there is no significant structural difference between the hRANKL-mRANK and mRANKL-mRANK complexes. The critical residues in mRANKL involved in the mRANKL-mRANK interactions such as His-179, Lys-180, Gly-191, Arg-222, Glu-225, Asn-266, Glu-268, Lys-281, Arg-283, Asp-299 and Asp-301 are all conserved in hRANKL and make similar interactions to mRANK (Fig. 1B). Residues Arg-222 and Asp-299 in mRANKL, which make salt bridges to Asp-94 and Lys-97 of mRANK respectively, have been demonstrated to be critical for ligand-receptor interaction^29^. The binding affinities of Arg222Ala and Asp299Ala mRANKL mutants to the receptor are dramatically decreased according to Biacore analysis, and both mutants have completely lost their ability to promote functional osteoclast formation^29^. For the hRANKL-mRANK complex, the corresponding residues (Arg-223 and Asp-300) in hRANKL form similar salt bridges with Asp-94 and Lys-97 in mRANK (Fig. 1C), indicating its important role in hRANKL-mRANK interaction.

### hRANKL mutants without osteoclast-stimulating activity

**Figure 2:**
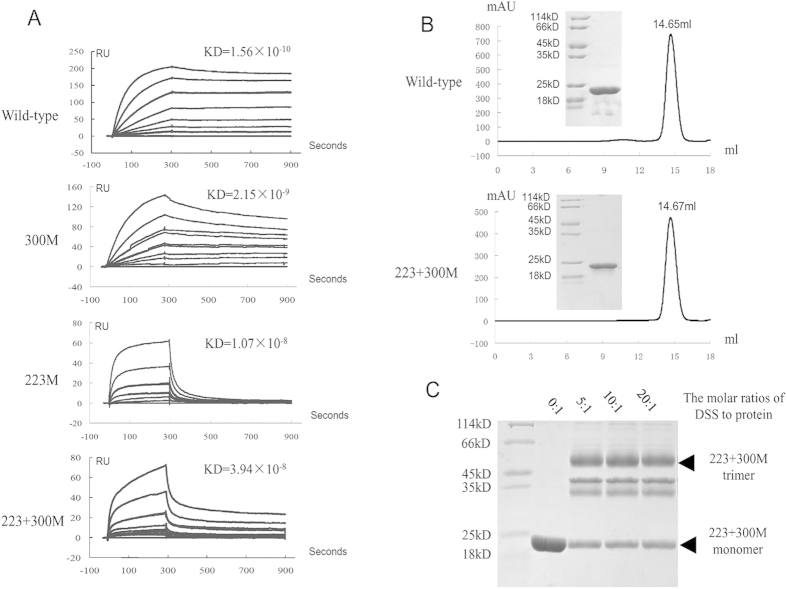
The effect of double-site mutation of hRANKL on receptor binding. (**A**) The affinities of wild-type hRANKL, two single-site mutants (300 M and 223 M) and a double-site mutant (223 + 300 M) for binding to mRANK were measured by Biacore analyses using a chelating NTA sensor chip. Recombinant TNFRSF9 was used as a negative control. Different concentrations of hRANKL or its mutants (0, 0.47, 0.94, 1.88, 3.75, 7.5, 15 and 30 nM) were injected into channels and signals recorded as sensorgrams. The equilibrium-dissociation constants were calculated using BIAevaluation software 4.1. (**B**) The purity of wild-type hRANKL and its double-site mutant (223 + 300 M) were analyzed by gel filtration chromatography and 12% SDS-PAGE. (**C**) The trimeric form of hRANKL double-site mutant (223 + 300 M) was demonstrated by the cross-linking assay. The protein in PBS buffer (pH 7.3) were mixed with different amounts of DSS on ice for 1 hours on ice and terminated with 50 mM Tris (pH 7.5).

**Figure 3:**
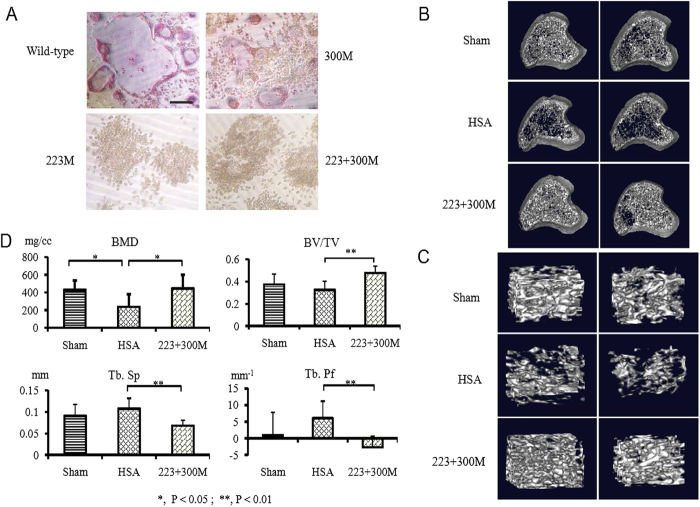
Evaluation of bone-resorption inhibition effect of 223 + 300 M protein immunization on OVX rats using micro-CT. (**A**) The osteoclast maturation-inducing abilities of hRANKL and its mutants were examined by RAW264.7 cells differentiation experiment. RAW264.7 cells were cultured in the presence or absence of 50 ng/ml hRANKL or its mutants for 4 days. Cells were fixed and stained for TRAP. Bar, 200 μm. OVX rats were immunized with 223 + 300 M protein and HSA, which served as a negative control. Sham-operated rats were used as a positive control. Three-dimensional images of (**B**) proximal tibia slices and (**C**) trabecular bone architectures of volume of interest (VOI) extracted from proximal tibia are exhibited. Scanning for the proximal tibias was initiated proximally at the level of growth plate and the resolution was 19 μm in all three spatial dimensions. Two representative images in each group were shown. (**D**) Bone Mineral Density (BMD) and other morphometric and nonmetric parameters including Bone Volume/Total Volume (Bv/Tv), Trabecular Spacing (Tb.Sp) and Trabecular Pattern Factor (Tb.Pf) are quantitatively analyzed. Data are shown as mean ± SD. Student’s *t-*test was used to evaluate statistically significant differences between the values.

Based on the structure of the hRANKL-mRANK complex we designed hRANKL mutants with abrogated receptor binding ability. Considering the important roles of Arg223 and Asp300, a set of hRANKL mutants, including Arg223Ala (223 M), Asp300Ala (300 M) and Arg223Ala plus Asp300Ala (223 + 300 M) were generated to screen for hRANKL mutants that lack osteoclast-stimulating activity. Biacore analyses showed that hRANKL has a comparable binding affinity for mRANK (K_d_ 1.56 × 10^−10^ M) to mRANKL (K_d_ of 6.8 × 10^−11^ M) (Fig. 2A). Mutants 223 M and 300 M exhibited 20-fold and 70-fold decreased affinity mRANK while the double mutant 223 + 300 M exhibited a larger, 260-fold, decrease in K_d_. The function and biochemical properties of mutant 223 + 300 M were further investigated. Gel filtration chromatography showed that the elution volume for 223 + 300 M was 14.67 ml, equal to 14.65 ml of wild-type hRANKL (Fig. 2B). Chemical cross-linking demonstrated that the 223 + 300 M recombinant protein, similar to wild-type hRANKL, was trimeric (Fig. 2C). Subsequently the osteoclast-stimulating activity of 223 + 300 M was examined by RAW264.7 cell differentiation experiments. The results showed, contrary to the strong osteoclast maturation-inducing ability of wild-type hRANKL, almost no matured osteoclast was formed after incubation with 223 + 300 M, indicating that this mutant has lost its osteoclast-stimulating activity (Fig. 3A).

### Bone-resorption inhibition effect of 223 + 300 M protein immunization on OVX rats

Since 223 + 300 M mutant lacks osteoclast-stimulating activity, immunization with this protein might induce anti-RANKL antibodies that would inhibit the osteoclast-stimulating activity of endogenous RANKL. To demonstrate this possibility, OVX rats were used as an osteoporosis animal model to examine the therapeutic effect of 223 + 300 M protein immunization on osteoporosis. In OVX rats immunized by hRANKL mutant, the tibias were scanned by high-resolution micro-computed tomography (micro-CT). Representative three-dimensional images of proximal tibia slices and the trabecular bone architecture of volume of interest (VOI) extracted from proximal tibia were shown in Fig. 3B,C. Compared to sham-operated rats, the plate-like structures of trabecular bone from OVX rats immunized with HSA are more often fenestrated and the rods forming the trabecular network became thinner until they disappear, leaving the structure less well connected, indicating the occurrence of osteoporosis after ovariectomy. Similar to normal control rats, the trabecular bones from OVX rats immunized with 223 + 300 M, however, were significantly thicker and denser than those from OVX rats immunized with HSA, and similar to normal control rats. In addition to the visual assessment, the bone mineral densities (BMD) of tibial trabecular bones, defined as the volumetric density of calcium hydroxyapatite and used as an indirect indicator of osteoporosis in clinical medicine, were analyzed by a quantitative micro-CT. The analysis showed that the BMDs of both the Sham group (431.1 ± 104.8 mg/cc) and the 223 + 300 M group (445.8 ± 156.9 mg/cc) were significantly higher than that of the HSA group (238.0 ± 139.1 mg/cc, both P < 0.05) (Fig. 3D). Besides, morphometric and nonmetric parameters including the bone volume density (Bv/Tv), which is a measure of the volume of the bone trabeculae relative to the total volume of the VOI, the trabecular spacing (Tb.Sp) and the trabecular pattern factor (Tb.Pf) were evaluated individually by quantitative micro-CT (Fig. 3D). There was a discrepancy in the variation of morphometric and nonmetric parameters between two OVX rat groups, one immunized with HSA and the other immunized with 223 + 300 M. These discrepancies were more pronounced and had statistical significances (P < 0.01). As expected, the values of Bv/Tv, Tb.Sp and Tb.Pf of sham group and OVX 223 + 300 M group remained the similar level although there were some experimental variations. These results clearly demonstrated a therapeutic effect of 223 + 300 M protein as an active immunization agent on osteoporosis.

### Anti-RANKL antibodies induced by 223 + 300 M protein immunization inhibit non-human RANKL-mediated osteoclast differentiation

**Figure 4:**
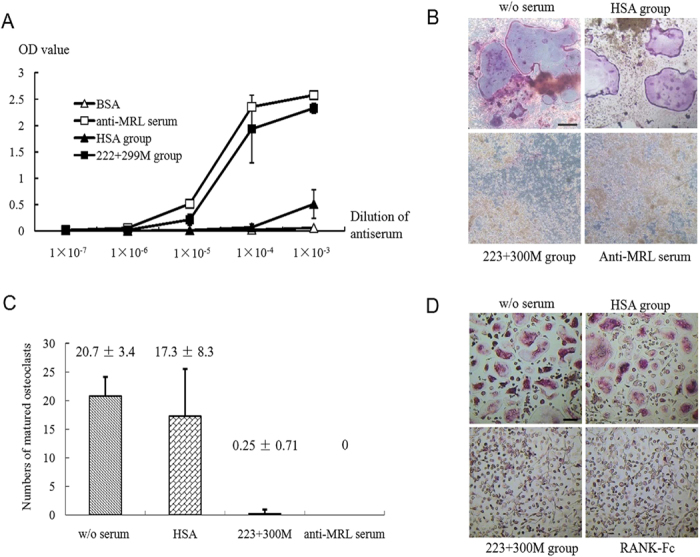
Examination of anti-RANKL antibodies in sera of OVX rats after protein immunization. (**A**) the titer of antisera against mRANKL was test by ELISA. The mRANKL protein and BSA serving as a negative control were coated onto ELISA plates. Anti-HSA sera, anti- 223 + 300 M sera obtained from immunized OVX rats, and rabbit anti-mRANKL serum (anti-MRL serum) which served as a positive control, were added at the different dilutions. The values of the Optical Density (O.D.) at 450 nm were used to evaluate the sensitivities of antisera. Data are shown as mean ± SD. The inhibition effects of antisera on mRANKL-induced BMM cell differentiation (**B**) and on hRANKL-induced human CD14^+^ cell differentiation (**D**) were examined. Cells were fixed and stained with TRAP kit. Bar, 200 μm and 50 μm individually. (**C**) The numbers of matured osteoclast cells (TRAP staining-positive, multinucleated) in (**B**) per well were scored and expressed as mean ± SD.

We then examined if 222 + 300 M protein immunization induces the production of anti-RANKL Abs against heterologous RANKL besides hRANKL. Considering ~98% identity of the RANKL ectodomain (residues 163–318 of rat RANKL, and residues 161–316 of mRANKL), and only one residue difference (Ala^123^ of rat RANK, and Ser^123^ of mRANK) in CRD2 and CRD3 regions of RANK, which involved in direct contacts with the ligands^29^, between *Rattus norvegicus* and *Mus musculus*, mRANKL and mouse-derived cells instead of rat RANKL and rat-derived cells were used for further analyses. Elisa analysis indicated that anti-sera obtained from rats immunized with 223 + 300 M protein could detect mRANKL with high titer compared with anti-mRANKL anti-sera, the positive control, and anti-sera obtained from rats immunized with HSA, the negative control (Fig. 4A). Osteoclast differentiation experiments with mouse bone marrow-derived macrophages showed that antisera obtained from rats immunized with HSA didn’t inhibit osteoclast differentiation stimulated by mRANKL, while anti-sera obtained from rats immunized with 223 + 300 M protein successfully abolished the differentiation (Fig. 4B,C). Meanwhile, anti-sera obtained from rats immunized with 223 + 300 M protein also inhibit osteoclastic differentiation of human CD14^+^ cells stimulated by hRANKL (Fig. 4D).

## Discussion

Osteoclasts are unique in their ability to resorb bone and plays important roles in excessive bone resorption in pathological situations such as osteoporosis or arthritis. RANKL-RANK signaling regulates the development and function of osteoclast and their interaction provides potentially an ideal target for intervention for osteoporosis and other diseases associated with bone loss. A successful example is the application of the fully humanized anti-RANKL mAb, Denosumab in clinical therapies of osteoporosis and bone loss caused by metastatic prostate or breast tumor, where the antibody acted as an inhibitor of RANKL-RANK pathway. The RANKL-RANK pathway is also involved in pathological process of atherosclerosis and progestin-driven mammary cancer. Therefore designing and validating a novel RANKL-RANK pathway inhibitor is also valuable for some non-bone related illnesses.

In recent years, active anti-cytokine immunotherapy has been proved to be an effective way to treat several inflammatory disorders associated with overproduced or abnormally released cytokines such as TNF-a, IFN-a, IL-4 and VEGF. The common strategy currently is to use human origin cytokines as antigen, to induce an anti-self immune response. To overcome self-tolerance a hertogenous and immune active carrier incorporated into cytokine interest including KLH protein^37^, virus like particle^38^, or built-in T cell epitope is used^39^. Recently the results of a phase II clinical trial with TNF-KLH vaccine show that TNF-K therapeutic vaccination induced dose- and schedule- dependent anti-TNF antibodies in RA patients and was well tolerated^40^. Patients who developed anti-TNF antibodies showed a trend toward clinical improvement. Further studies are required to demonstrate the clinical benefit of the strategy. It is notifying, there are several disadvantages associated with this strategy: 1, the structure of the cytokine would change after modification and the antibodies induced might not be the “correct” antibody to neutralize the desired toxic cytokine. 2, undesired immune responses produced against the carrier protein might cause unexpected side-effects. 3, administration of auto-cytokine with remained activity may make the disease situation worse. To circumvent the problems we attempt a strategy to use a cytokine from a different species as an immunogen to bypass immune tolerance toward a self-protein. To avoid the immunogen acting as a cytokine we introduced mutations to disrupt its interaction with its target.

As a proof of concept, immunization of rat model with recombinant hRANKL was successful in the prevention of osteoporosis. hRANKL, has been demonstrated to bind xenogeneic receptor such as mRANK and induce non-human osteoclast maturation by Biacore analysis and RAW264.7 cells differentiation experiment. To circumvent the problem of the immunogen having cytokine activity mutants of hRANKL were generated to demolish its interaction to mRANK based on the structure of the complex. The RAW264.7 cells differentiation experiment indicated that the mutant 223 + 300 M abolished osteoclast-stimulating activity entirely. Visual assessment of trabecular bone architecture and quantitative analyses of BMD and several morphometric and nonmetric parameters demonstrated the treatment effect of the protein immunization on osteoporosis in an OVX rat model. The antisera obtained from rats immunized with hRANKL could detect mRANKL, which share ∼90% identity with hRANKL in the ectodomain region and ∼98% identity with rRANKL, with high titer and almost entirely abolish the osteoclast differentiation of mouse bone marrow-derived macrophages. Spohn G *et al.* reported a therapeutic vaccine approach for osteoporosis by active immunization of OVX mice with RANKL covalently linked to virus-like particles (VLP)^41^. However, there was a concern in their therapeutic approach: as shown in their report, RANKL-VLP was able to efficiently induce osteoclast formation from bone marrow cells^41^. The injected therapeutic reagent may therefore deteriorate osteoporosis and other RANKL-stimulating bone related and irrelated illnesses. This problem was solved in our treatment strategy by using RANKL mutant without osteoclast-stimulating activity. Even if the injected protein didn’t induce a sufficiently effective immune response to meet the therapeutic requirement, which is harmless for receivers. Similarly a human IL-5 produced the antibody in murine disease model to exhibit therapeutic effects, whereas the autologous murine protein did not^42^. A group of mutated proteins derived from IL-6 cytokine was used to induce active immunization against IL-6^43^.

In this manuscript we have demonstrated that immunization with a RANKL mutant generates inter-species anti-RANKL antibody, which blocks interaction between RANKL and its receptor and further prevents the formation of the osteoclast and improves the density of the bone in the rats with ovariectomy. However, further experiments are required to assess whether that mechanical performances of the bones are improved, and older animals would be used in future tests to eliminate the potential effect of ovariectomy on skeletal acquisition. Besides, we need to address in further experiments if the strategy can be used in osteoporosis therapy for human beings. For example, a mouse origin RANKL mutant would be required to test its ability to induce antibodies against RANKL in different animals before starting a clinical trial. We are testing the idea with rabbits and the primary results are encouraging (data not shown). The positive results will pave the way for creating a novel therapy for osteoporosis.

In summary we have adopted an active anti-RANKL immunotherapy strategy using inter-species RANKL mutant for osteoporosis in an OVX rat model. Our studies indicate active anti-RANKL immunotherapy using inter-species RANKL mutant could be a useful strategy for human osteoporosis and provide a new concept for general active immunization protocol for anti-cytokine therapy.

## Materials and Methods

### Cell Lines, mouse Strains and reagents

Raw264.7 cells were purchased from the American Type Culture Collection and propagated in culture according to the manufacturer’s protocol. Bone marrow-derived macrophages (BMMs) were isolated from bone marrow cells which were obtained by flushing femurs and tibiae from 7-wk-old BALB/c mice as reported^44^. Female Sprague–Dawley rats aged 2 months were purchased from Vital River Laboratories, Beijing, China. Oligonucleotides were synthesized by Sangon Biotech (Shanghai, China). Restriction enzymes and T4 DNA ligase were purchased from Fermentas (Burlington, Ontario, Canada). Pfu DNA polymerase was purchased from Tiangen Biotech (Beijing, China). Reduced Glutathione and TRAP kit were purchased from Sigma-Aldrich (St. Louis, MO).

### Cloning, protein expression, and purification

The preparation of the expression plasmids for the ectodomain of human RANKL (residues 158–317) with a N-terminal GST tag, pGEX-6P-hRANKL, and the extracellular domain of mouse RANK, pET28a-mRANK, were reported previously^29^,^45^. Site-directed mutageneses of *hrankl* were performed using QuickChange Kit supplied by Stratagene (Agilent Technologies, Palo Alto, CA). The mutants were verified by DNA sequencing. The mRANK protein was expressed and purified as reported^29^. Briefly, BL21 (TransGen, Beijing, China) with pGEX-6P-hRANKL and pGEX-6P-hRANKL mutants were grown in LB medium and induced with IPTG (0.5 mM) overnight at 20 °C. The soluble extracellular domain of human RANKL and its mutants were expressed as a GST fusion protein which were purified with glutathione-Sepharose fast flow 4B beads (GE Healthcare) according to the manufacturer’s protocol and the tags were cleaved with PreScission protease (GE healthcare). The cleaved human RANKL proteins were further purified by size exclusion chromatography (Superdex 200) in phosphate-buffered saline, pH 7.4 (PBS).

### Crystallization, data collection and structure determination

Purified hRANKL and mRANK both in 0.1 M Tris solution at pH 7.0 were mixed at 1:1 molar ratio and concentrated to 10 mg/mL. Crystallization were carried out at a temperature of 294 K using nano-litre sitting drop vapour diffusion in the crystallization facility of the OPPF^46^. Crystals of hRANKL-mRANK were grown by microseeding in the same condition as those of mRANKL-mRANK complex, which contains 0.1 M sodium di-hydrogen phosphate, 2 M sodium chloride, 0.1 M potassium di-hydrogen phosphate and 0.1 M MES (pH 6.5)^47^. Crystal seeds were prepared by crushing mRANKL-mRANK crystals.

X-ray diffraction data were collected at beamline ID23-2 at the ESRF (Grenoble, France). A total of 225 images of 1.0° oscillation were collected from 4 positions of a single crystal at a wavelength of 0.872 Å. 70% reservoir solution and 30% glycerol were mixed and was then added to the crystallization drops as cryo-protectant. Crystals were frozen and maintained at 100 K by a stream of nitrogen gas during data collections. Data images were indexed, integrated and merged using HKL2000^48^.

The space group of the hRANKL-mRANK complex crystals is *P6*_*3*_ with unit cell dimensions *a* = *b* = 122.2 Å, *c* = 94.5 Å. There is one hRANKL and one mRANK molecules in the crystallographic asymmetric unit, which are assembled to form the biological hetero-hexameric complex through the 3-fold crystallographic symmetry. The orientation and position of the complex was determined using the mRANKL-mRANK complex^49^ as a search model for molecular replacement with MOLREP^50^ and the structure was refined with REFMAC^51^. The final refined model has an R-factor of 0.165 (R-free: 0.204) to 2.8 Å resolution with rmsds of 0.007 Å for bond lengths and 1.4° for bond angles from ideal values. The statistics for x-ray data and structure refinement are given in Table 1.

The coordinates and structure factors have been deposited with the RCSB under accession code: 5BNQ.

### Cross-linking assay

Different amounts of the chemical cross-linking reagent, disuccinimidyl suberate (DSS) purchased from Pierce (Rockford, IL), and the hRANKL double-site mutant (223 + 300 M) were mixed (the molar ratios of DSS to protein are 0:1, 5:1, 10:1 and 20:1) and the mixture was kept on ice for 1 hour for interaction which was terminated with 50 mM Tris (pH 7.5). Then the trimeric form of 223 + 300 M was demonstrated with SDS-PAGE.

### Treatments of animals

All animal studies were carried out in accordance with the approved guidelines of the Institutional Animal Care and Use Committee of IMCAS. Twenty-four Sprague Dawley rats were divided equally into three groups: the negative control group was given bilateral OVX and immunized with Human Serum Albumin (HSA); the positive control group was given the sham operation; the treatment group was given OVX and immunized with 223 + 300 M. The protein used for immunization was mixed with aluminium hydroxide adjuvant. Three months after the surgery, the animals received the first immunization. The interval of each immunization was two weeks and the amount of protein for immunization was 0.2 mg each time per rat. Each rat received six immunizations in total. Six months after the first immunization the rats were sacrificed for μ-CT scanning and the sera were collected for ELISA to determine the titer of anti-RANKL antibodies. After the blood was collected, the tibiae were removed. Adherent soft tissue was cleaned off and bone specimens were subjected to micro-CT analysis.

### Micro-CT scanning and data analysis

The right proximal tibiae were scanned with Quantum FX μ-CT (Perkin Elmer) at an energy level of 90 kV and intensity of 0.16 mA with a field of view of 10 mm (voxel size, 20 μm; scanning time, 3 min). Scanning for the proximal tibia was initiated from the level of knee joint cavity to tibia distally for 512 slices totally. Evaluations were performed on 100 slices beginning from the slice which was defined as the plane where the growth plate had just disappeared at the cross-section. Bone morphometric measurements, including Bone Mineral Density (BMD, mg/cc), Bone Volume/Total Volume (Bv/Tv), trabecular separation (Tb.Sp, mm) and Trabecular Pattern Factor (Tb.Pf, mm^−1^) were calculated by the software Inveon research workplace. The value of bone morphometric parameters were shown as mean ± SD. Student’s *t-*test was used to evaluate statistically significant differences between the values. A *p* value less than 0.05 was considered statistically significant and correlations were considered high significance at the *p* value less than 0.01 level.

### Surface plasmon resonance

The affinities of wild-type hRANKL, two single-site mutants (300 M and 223 M) and a double-site mutant (223 + 300 M) binding to mRANK were measured on a Biacore 3000 (GE Heathcare). A NTA sensor chip (GE Heathcare) was charged by 0.3 M NiSO_4_, and then mRANK (50 nM, 15 ul) injected into the channel to load. hRANKL and its mutants in different concentrations (0, 0.47, 0.94, 1.88, 3.75, 7.5, 15 and 30 nM) were injected into the channels while the signals were recorded as sensorgrams. Recombinant TNFRSF9 with two His-tags was injected into a different channel as a control. BIAevaluation software 4.1 was used to calculate the equilibrium-dissociation constants (K_d_).

### Osteoclast formation and tartrate-resistant acid phosphatase staining

RAW264.7 cells were used to evaluate whether hRANKL and its mutants could induce RAW264.7 cells into mature osteoclast-like cells. RAW264.7 cells were cultured in the α-MEM media containing 10% fetal bovine serum (FBS) and 50 ng/ml hRANKL or its mutants for 4 days. Then the cells were fixed and stained with TRAP kit.

Sera from sham operation rats, 223 + 300 M immunized rats and HSA immunized rats were diluted a hundredfold with α-MEM media containing 10% fetal bovine serum (FBS) and 50 ng/ml mRANKL. BMMs were seeded into 4 wells for each group at a concentration of 5 × 10^5^ cells/well in a 24-well culture plate. After 3 days, the media were changed and then the media were changed at an interval of 2 days for 3 times. The tartrate-resistant acid phosphatase staining was executed using TRAP kit after 2 days of the last changing of media. The numbers of TRAP staining-positive, multinucleated (>3) cells per well were counted under a light microscope. Data were shown as mean ± SD.

### Determination of the titer of antisera against mouse RANKL by ELISA

The mRANKL protein and BSA serving as a negative control were coated 1 μg/well in PBS onto ELISA plates overnight. Anti-HSA sera, anti-223 + 300 M sera obtained from immunized OVX rats, and rabbit anti-mRANKL serum which served as a positive control, were added at the different dilution times from 10^3^ to 10^7^. Each sample had double-pored wells. The values of the Optical Density (O.D.) at 450 nm were used to evaluate the sensitivities of antisera. Data were shown as mean ± SD.

### Human osteoclast formation assay

All experiments for human PBMCs were carried out in accordance with the approved guidelines of the Institutional Human Ethics Committee of IMCAS. CD14^+^ cells were isolated from PBMC by Magnetic Cell Sorting (MACS) and seeded at a density of 5 × 10^4^ cells per well in 96-well plates. The cells were cultured in 200 μl α-MEM media containing 10% fetal bovine serum (FBS), 33 ng/ml hM-CSF and 100 ng/ml hRANKL. The media was changed every two days. Seven days after the seeding of the cells, trap staining was performed. The sera collected from 223 + 300 M immunized rats and HSA immunized rats were diluted 200 times and added into wells seeded with CD14^+^ cells served as test groups and negative controls. The wells which were added into 2 μg/ml RANK-Fc were served as positive controls.

## Additional Information

**How to cite this article**: Liu, C. *et al.* A RANKL mutant used as an inter-species vaccine for efficient immunotherapy of osteoporosis. *Sci. Rep.* **5**, 14150; doi: 10.1038/srep14150 (2015).

## Figures and Tables

**Table 1:** X-ray data collection and refinement statistics.

Data collection details	
X-ray source	ID23-2, ESRF
Wavelength (Å)	0.8726
Space group	*P*6_3_
Unit cell (*a, b, c* [Å])	122.2, 122.2, 94.5
Resolution range^A^ (Å)	30.0 – 2.80(2.90-2.80)
Unique reflections	19916(1959)
Completeness (%)	99.8(98.2)
Redundancy	12.1(5.7)
Average *I/σI*	14.6(2.1)
*R*_*merge*_	0.197(0.818)
Refinement statistics:	
Resolution range (Å)	30.0–2.80
No. of reflections(working/test)	18883/1018
*R*-factor^B^(*R*_*work*_/*R*_*free*_)	0.165/0.204
No. of atoms(protein/water/other)	2505/79/29
Rms bond length deviation (Å)	0.007
Rms bond angle deviation (°)	1.4
Average B-factor(protein/water/other)	36/45/85

A. Numbers in the brackets are for the highest resolution shell. B. *R*_*work*_ and *R*_*free*_ are defined by 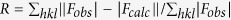, where *h, k, l* are the indices of the reflections (used in refinement for *R*_*work*_; 5%, not used in refinement for *R*_*free*_), *F*_*obs*_and *F*_*calc*_ are the structure factors, deduced from measured intensities and calculated from the model, respectively.
